# One-pot and one-step automated radio-synthesis of [^18^F]AlF-FAPI-74 using a multi purpose synthesizer: a proof-of-concept experiment

**DOI:** 10.1186/s41181-021-00142-z

**Published:** 2021-08-21

**Authors:** Sadahiro Naka, Tadashi Watabe, Thomas Lindner, Jens Cardinale, Kenta Kurimoto, Melissa Moore, Mitsuaki Tatsumi, Yuriko Mori, Eku Shimosegawa, Frank Valla, Hiroki Kato, Frederik L. Giesel

**Affiliations:** 1 Department of Nuclear Medicine and Tracer Kinetics, Osaka University Graduate School of Medicine, 2-2 Yamadaoka, Suita, Osaka 565-0871 Japan; 2 Department of Radiology, Osaka University Hospital, 2-15 Yamadaoka, Suita, Osaka 565-0871 Japan; 3 Department for Nuclear Medicine, University Hospital Heidelberg, INF 400, 69120 Heidelberg, Germany; 4 Department of Nuclear Medicine, University Hospital Düsseldorf, Düsseldorf, Germany; 5 SOFIE, 21000 Atlantic Boulevard Suite 730, Dulles, VA 20166 USA; 6 Department of Molecular Imaging in Medicine, Osaka University Graduate School of Medicine, 2-2 Yamadaoka, Suita, Osaka 565-0871 Japan; 7 Institute for Radiation Sciences, Osaka University, 2-2 Yamadaoka, Suita, Osaka 565-0871 Japan

**Keywords:** Positron emission tomography, Fibroblast activation protein, [^18^F]AlF-FAPI-74, Automated synthesis

## Abstract

**Background:**

Fibroblast activation protein (FAP) is overexpressed in the stroma of many types of cancer. [^18^F]AlF-FAPI-74 is a positron emission tomography tracer with high selectivity for FAP, which has already shown high accumulation within human tumors in clinical studies. However, [^18^F]AlF-FAPI-74 radiosynthesis has not been optimized using an automated synthesizer. Herein, we report a one-pot and one-step automated radiosynthesis method using a multi purpose synthesizer.

**Results:**

Radiosynthesis of [^18^F]AlF-FAPI-74 was performed using a cassette-type multi purpose synthesizer CFN-MPS200. After the recovery rate of trapped [^18^F]fluoride onto the anion-exchange cartridge using a small amount of eluent was investigated manually, a dedicated [^18^F]AlF-FAPI-74 synthesis cassette and synthesis program for one-pot and one-step fluorination was developed. The solutions for the formulation of [^18^F]AlF-FAPI-74 synthesized using this were evaluated to obtain stable radiochemical purity. The recovery rate of [^18^F]fluoride with only 300 µL of eluent ranged 90 ± 9% by introduction from the male side and elution from the female side of the cartridge. In automated synthesis, the eluted [^18^F]fluoride and precursor solution containing aluminum chloride were mixed; then, fluorination was performed in a one-pot and one-step process at room temperature for 5 min, followed by 15 min at 95 °C. As a result, the radioactivity of [^18^F]AlF-FAPI-74 was 11.3 ± 1.1 GBq at the end of synthesis from 32 to 40 GBq of [^18^F]fluoride, and its radiochemical yield was 37 ± 4% (n = 10). The radiochemical purity at the end of the synthesis was ≥ 97% for all formulation solutions. When the diluent was saline, the radiochemical purity markedly decreased after 4 h of synthesis. In contrast, with phosphate-buffered saline (pH 7.4) or 10 mM phosphate-buffered saline (pH 6.7) containing 100 mg of sodium ascorbate, the radiochemical purity was stable at 97%. Non-radioactive AlF-FAPI-74 and total impurities, including non-radioactive AlF-FAPI-74, were 0.3 ± 0.1 µg/mL and 2.8 ± 0.6 µg/mL. Ethanol concentration and residual DMSO were 5.5 ± 0.2% and 21 ± 6 ppm, respectively.

**Conclusions:**

We established a one-pot one-step automated synthesis method using a CFN-MPS200 synthesizer that provided high radioactivity and stable radiochemical purity for possible clinical applications.

## Background

The fibroblast activation protein (FAP) has already been shown to be expressed at low levels in normal tissues but is highly expressed in the stroma of many cancer types (Brennen et al. 2012; Hamson et al. 2014). It has already been reported that compounds with high selectivity for FAP, such as FAPI-02, FAPI-04, and FAPI-46, are useful for diagnosis using labeled positron emitters (^64^Cu or ^68^ Ga) and treatment with labeled alpha or beta emitters (^64^Cu, ^225^Ac, ^90^Y, or ^153^Sm) for cancer, namely theranostics (Giesel et al. 2019; Kratochwil et al. 2019; Watabe et al. 2020; Kratochwil et al. 2021). Among these, [^68^Ga]FAPI-46 has already established automation using a synthesizer for clinical application; however, ^68^Ga used for radio-labeling was supplied by a ^68^Ge/^68^Ga generator (Spreckelmeyer et al. 2020). Therefore, the radioactivity obtained is low (700–1700 MBq at the end of synthesis [EOS]) and, as its half-life is 68 min, the number of positron emission tomography (PET) scans per day is thus limited, and delivery to remote areas is also difficult.

FAPI-74 has recently attracted attention as a target for theranostics. [^18^F]AlF-FAPI-74 and [^68^Ga]FAPI-74 have already shown high accumulation within human tumors in clinical studies (Giesel et al. 2021). FAPI-74 is a compound with 1,4,7-triazacyclononane-N, N',N'-triacetic acid (NOTA) bound as a chelating agent, which enables the labeling of ^68^Ga and ^18^F via a complex with aluminum ([^18^F]AlF), as previously reported (McBride et al. 2009; Malik et al. 2015; Boschi et al. 2016; Allott et al. 2017; Kersemans et al. 2018; Tshibangu et al. 2020). When obtaining a large amount of [^18^F]AlF-FAPI-74 using [^18^F]AlF, automated synthesis is essential for radiation protection; however, this has not been optimized with a small amount of reaction solution using an automated synthesizer. The important points for transferring the procedure optimized by Giesel et al. for manual synthesis to the automated synthesizer were: (1) the collection procedure of produced [^18^F]fluoride into the reactor with a high recovery rate using a small amount of eluent (300 µL), (2) the method of adding a small amount of reagent (4–300 µL) for fluorination and the fluorination procedure simplify, and (3) the composition of the formulation solution to obtain stable radiochemical purity (RCP) at high radioactivity to ensure that sufficient time is available for in-house clinical research (4 h following the synthesis) (Giesel et al. 2021). In 1) of important point, since it has been reported that elution of [^18^F]fluoride from QMA column is insufficient at an amount of 300 µL (Kersemans et al. 2018), we need to select the type of QMA column, the sorbent amount, and the direction of introduction and elution into the column with reference to the back-flush protocol used by other study (Zlatopolskiy et al. 2015).

In this study, we report the establishment of a one-pot and one-step automated radiosynthesis method using a multi purpose synthesizer CFN-MPS200 (Sumitomo Heavy Industries, Tokyo, Japan) that achieves the above-mentioned important points.

## Methods

### General

For the production of [^18^F]fluoride, CYPRIS HM-18 manufactured by Sumitomo Heavy Industries was used, and [^18^O]H_2_O as the target water was purchased from Rotem (> 98 atom%, Mishor Yamin, Israel). The Cupid System (Sumitomo Heavy Industries, Tokyo, Japan) was also used to control the CFN-MPS200 in the automated synthesis of [^18^F]AlF-FAPI-74 and N_2_ gas (> 99.9999%) supplied to synthesizer was purchased from Taiyo Nippon Sanso (Tokyo, Japan).

**Fig. 1:**
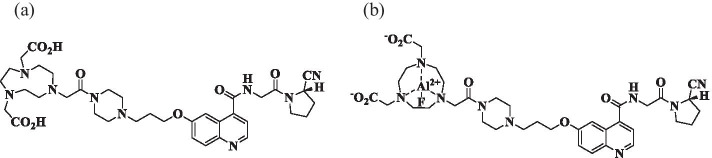
Structures of the FAPI-74 precursor and the reference standard of AlF-FAPI-74. **a** The precursor was bound 1,4,7-triazacyclononane-N, N′,N′-triacetic acid (NOTA) as a chelating agent. **b** The reference standard of AlF-FAPI-74

The reagents used for the synthesis of [^18^F]AlF-FAPI-74 were purchased from the following suppliers; ultrapure water (ultrapure grade) and ethanol (trace analysis grade) from Kanto Chemical (Tokyo, Japan), dimethyl sulfoxide (DMSO, Molecular Biology grade) from FUJIFILM Wako Pure Chemical (Osaka, Japan), aluminum chloride hexahydrate (AlCl_3_ 6H_2_O, ReagentPlus®) from Sigma-Aldrich (St. Louis, MI, USA), ascorbic acid (Biotechnology grade) from Nacalai Tesque (Kyoto, Japan), sodium ascorbate (USP grade) from Spectrum (New Brunswick, NJ, USA) and phosphate-buffered saline (pH 7.4) from ABX (Radeberg, Germany). These reagents were used without purification. Water for injection (WFI) and isotonic sodium chloride solution (saline) were all Japanese Pharmacopeia grade and were purchased from Otsuka Pharmaceutical (Tokyo, Japan). FAPI-74 precursors and a reference standard for [^18^F]AlF-FAPI-74 were provided by SOFIE (Dulles, VA, USA) (Fig. 1). Sep-Pak® Accell™ Plus QMA Plus Light cartridge (130 mg sorbent per cartridge), Sep-Pak® Accell™ QMA carbonate Plus Light Cartridge (46 mg sorbent per cartridge), and Oasis® Light HLB cartridges were purchased from Waters (Milford, MA, USA). For the quality control, high-performance liquid chromatography (HPLC) column and thin layer chromatography (TLC) plate were made by Merck (Kenilworth, NJ, USA).

### ***Recovery of [***^***18***^***F]fluoride from QMA cartridges (manual operation)***

Two types of QMA cartridges were preconditioned with 10 mL of 0.5 M sodium acetate buffer (pH 3.95 ± 0.05) and 20 mL of WFI. [^18^F]Fluoride was obtained by irradiating the proton beam to [^18^O]H_2_O using an 18 MeV energy cyclotron. After diluting part or all of the target water, containing [^18^F] fluoride, with 1–6 mL of WFI, 1–2 mL (ca. 20–1600 MBq) was introduced from the male or female side of the QMA cartridge. Then, 0.3 mL of 0.5 M sodium acetate buffer (pH 3.95 ± 0.05) was passed through the male or female side of the cartridge to recover [^18^F]fluoride. The recovery rate was calculated from the introduced radioactivity from male or female side (sum of the radioactivity that was retained in the QMA cartridge and the radioactivity that passed through it) and the radioactivity recovered from the male side or female side of the QMA cartridge.

### ***Radio-synthesis of [***^***18***^***F]AlF-FAPI-74 with the automated-synthesizer***

**Fig. 2:**
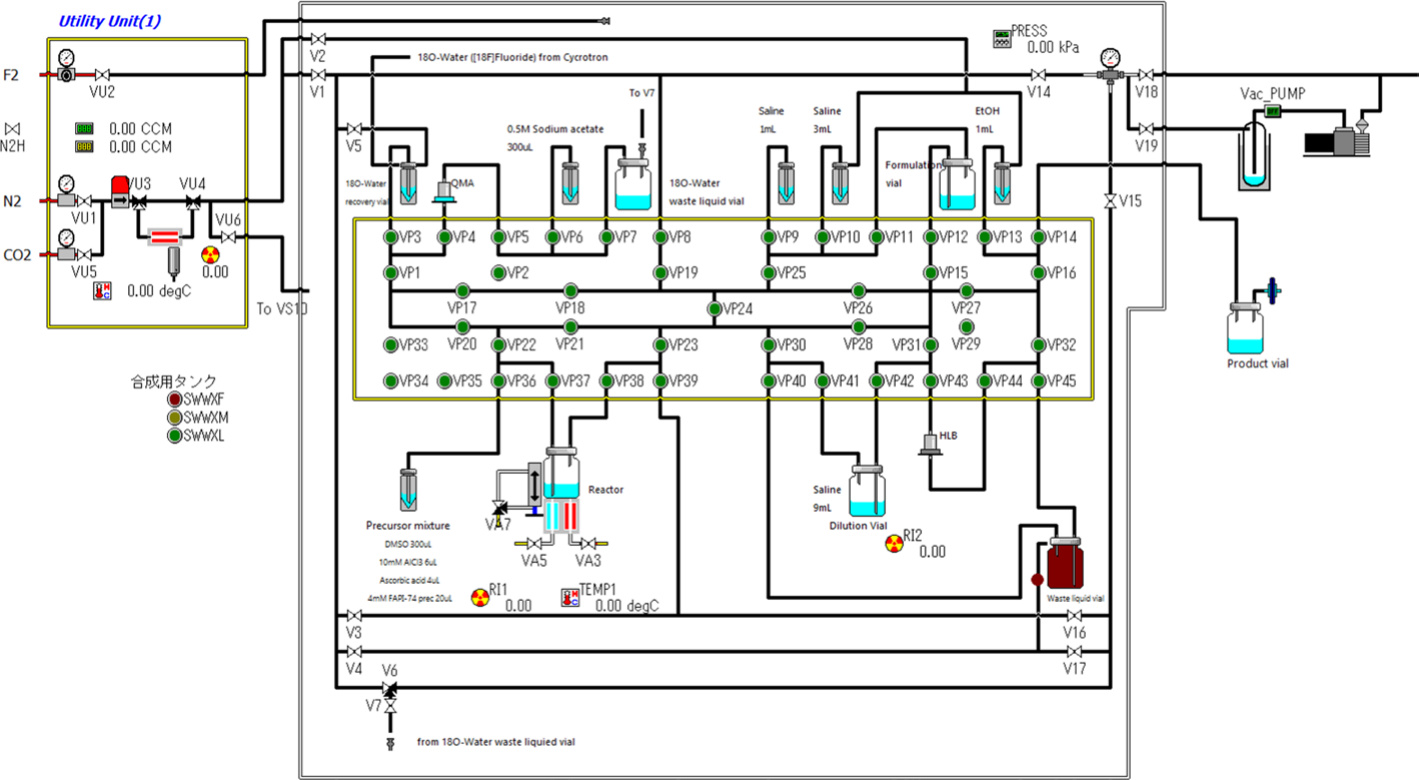
Systematic diagram of [^18^F]AlF-FAPI-74 with cupid system

For the synthesis of [^18^F]AlF-FAPI-74 on CFN-MPS200, previously known conditions have been adapted (Giesel et al. 2021). An automated synthesizer for the radiosynthesis of [^18^F]AlF-FAPI-74 was used as a single-use cassette-type CFN-MPS200 controlled by the Cupid System. Silicon was used as the material for the cassette, and a new systematic diagram was created specifically for [^18^F]AlF-FAPI-74 synthesis (Fig. 2). The process is described as follows.

**Fig. 3:**
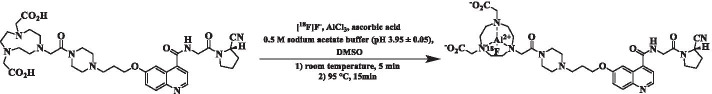
Reaction schema of [^18^F]AlF-FAPI-74 with one-pot and one-step radio-synthesis

[^18^F]Fluoride was recovered from the cyclotron to an ^18^O-water recovery vial and then loaded onto a QMA cartridge preconditioned with 10 mL of 0.5 M sodium acetate buffer (pH 3.95 ± 0.05) and 20 mL of ultrapure water from the cartridge male side via VP3 and VP4 under vacuum and 50 mL/min N_2_ gas and discarded to ^18^O-water waste liquid vial via VP5 and VP7. The [^18^F]fluoride on QMA cartridge was eluted with 300 µL of 0.5 M sodium acetate buffer (pH 3.95 ± 0.05) from the female cartridge side via VP6 and VP5 using a vacuum and collected into a glass reactor (Nichiden-Rika Glass, Hyogo, Japan) via VP4 to VP37. Further, the precursor mixture for one-step fluorination was added into the reactor via VP36 and VP37 using vacuum and then mixed with 50 mL/min N_2_ gas. The precursor mixture was composed of 300 µL of DMSO, 6 µL of 10 mM aluminum chloride aq. solution, 4 µL of 20% w/v ascorbic acid (aq.) solution, and 20 µL of 4 mM FAPI-74 precursor aq. solution. The radio-fluorination was carried out continuously for 5 min at room temperature, followed by 15 min at 95 °C with a closed system (Fig. 3). After cooling the reactor, the reaction mixture was transferred to a dilution vial where 9 mL of saline was added beforehand via VP37 to VP42 using 100 mL/min N_2_ gas flow. To collect the remaining radioactivity in the reactor and transfer line, 1 mL of saline was added to the reactor via VP9 to VP37 under vacuum and then transferred to the dilution vial via the same route. The dilution solution was passed through the HLB cartridge preconditioned with 5 mL ethanol and 20 mL of ultrapure water via VP42 and VP43 with 50 mL/min N_2_ gas flow, then discarded to waste liquid vial via VP44 and VP45. The HLB cartridge was washed with 3 mL of saline via VP10 to VP43 with 50 mL/min N_2_ and discarded to waste liquid vial via VP44 and VP45. Trapped [^18^F]AlF-FAPI-74 onto the HLB cartridge was collected into a formulation vial containing 14 mL of formulation solution using 1 mL of ethanol with 50 mL/min N_2_ gas flow via VP13 to VP44 and VP43 to VP12. This time, seven different formulation solutions containing saline were tested to find a composition that would prevent radiolysis and would be stable until 4 h after the synthesis: saline, saline containing 10 mg ascorbic acid and 90 mg sodium ascorbate (pH 5.0), saline containing 100 mg sodium ascorbate, commercially available phosphate-buffered saline (pH 7.4), phosphate-buffered saline (pH 7.4) containing 100 mg sodium ascorbate, 10 mM phosphate-buffered saline (pH 6.7), and 10 mM phosphate-buffered saline (pH 6.7) containing 100 mg sodium ascorbate.

### ***Quality control for [***^***18***^***F]AlF-FAPI-74***

All radioactivity, including [^18^F]AlF-FAPI-74, was measured using a dose calibrator. Measurement of the radiochemical purity, non-radioactive AlF-FAPI-74 (AlF-FAPI-74), and chemical impurities were carried out using an SPD-20A ultraviolet (UV) detector (λ = 264 nm) using a Shimadzu HPLC system and a Gabi-Star radioactivity detector (Elysia-Raytest, Straubenhardt, Germany). A Chromolith performance RP-18e (100 mm × 4.6 mm) as an analysis column was selected, and the mobile phase was acetonitrile (solvent A) and 0.1% trifluoroacetic acid (solvent B) in gradient mode; its condition was at 95% of solvent B from 0 to 3 min and then at 95% to 50% from 3 to 15 min. The method was calibrated using AlF-FAPI-74 as the reference standard (Linearity in the range from 0.05 to 20 µg/mL and the coefficient of determination of > 0.995 were confirmed). [^18^F]AlF-FAPI-74 was determined based on the retention time (RT) of the reference standard and the amount of carrier AlF-FAPI-74 calculated by calibration; the amount of all other chemical impurities was calculated based on the same calibration assuming similar extinction coefficients as AlF-FAPI-74. The set value of the column oven was 30 °C, and the flow rate was 2.0 mL/min.

Residual [^18^F]fluoride and [^18^F]AlF were analyzed using a radio-TLC analyzer, mini-Gita (Elysia-Raytest, Straubenhardt, Germany). The TLC plate used was HPTLC Silica gel 60 RP-18 and 1 v/v% phosphoric acid in saline/acetonitrile (50/50) as a developing solvent was filled in the tank for more than an h before the plate was deployed.

The residual DMSO and ethanol contents were measured using a flame ionization detector of a Shimadzu GC system. A DB-624 30 m × 0.32 mm, 1.8 µm (Agilent, Santa Clara, CA, USA) as an analysis column was selected and split injection mode (split ratio = 30:1). The carrier gas (helium) was 2 mL/min, and the column, injector, and detector temperatures were 40 °C for 5 min to 200 °C (20 °C/min) for 3 min, 200 °C, and 250 °C, respectively.

pH adjustment of buffers, etc., was performed using a pH meter with electrodes set for low volume (HORIBA, Kyoto, Japan), calibrated with pH standard buffer solution before use.

## Results

The recovery rate of [^18^F]fluoride from the QMA cartridge by manual operation is shown in Table 1. In both cartridges, the recovery rate of [^18^F]fluoride when input and output were introduced from the female side were 24% ± 6% and 23% ± 2%, respectively, and most of the radioactivity remained in the QMA cartridges (approximately 70%, data not shown). On the other hand, when input and output were introduced from the opposite side, especially when the input was male and the output was female, a high recovery rate of 90% ± 9% was obtained with a QMA cartridge (130 mg).

**Table 1:** Recovery of [^18^F]Fluoride from QMA cartridge under various conditions in manual operation

QMA types	Input of [^18^F]fluoride	Output of [^18^F]fluoride	Recovery rate (%)
QMA cartridge, 130 mg	Female to male	Female to male	24 ± 6 (n = 4)
	Female to male	Male to female	82 ± 24 (n = 5)
	Male to female	Female to male	90 ± 9 (n = 14)
QMA carbonate cartridge, 46 mg	Female to male	Female to male	23 ± 2 (n = 3)
	Male to female	Female to male	78 ± 7 (n = 3)

**Fig. 4:**
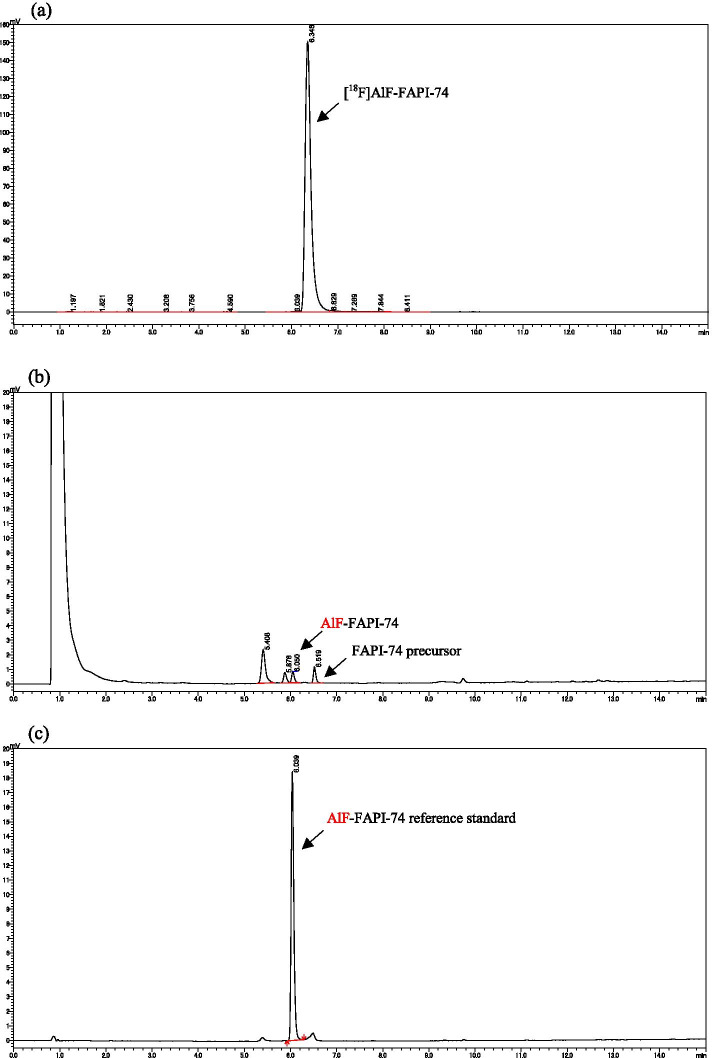
HPLC chromatograms of [^18^F]AlF-FAPI-74 with 10 mM phosphate buffered saline (pH 6.7) containing 100 mg sodium ascorbate as a formulation solution at the end of synthesis (EOS). **a** The typical radio-chromatogram of [^18^F]AlF-FAPI-74 solution. A similar chromatogram was shown in other formulation solutions and its radiochemical purity was ≥ 97%. **b** The typical ultraviolet (UV)-chromatogram (λ = 264 nm) of [^18^F]AlF-FAPI-74 solution. In the chromatogram, retention time (RT) 6.050 min was judged to be AlF-FAPI-74 based on the RT obtained from the AlF-FAPI-74 reference standard (**c**) and there is a time difference of approximately 0.3 min with the radioactivity detector. RT 6.519 min was judged to be FAPI-74 precursor based on prior analysis, and the remaining two peaks (RT 5.408 min and 5.878 min) were unknown impurity peaks. **c** The typical UV-chromatogram (λ = 264 nm) of AlF-FAPI-74 reference standard of 10 µg/mL (RT 6.039 min)

**Fig. 5:**
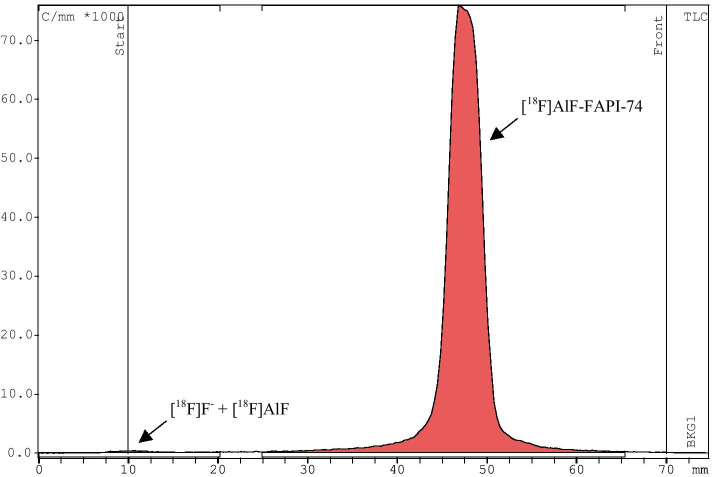
TLC chromatograms of [^18^F]AlF-FAPI-74 with 10 mM phosphate buffered saline (pH 6.7) containing 100 mg sodium ascorbate as a formulation solution. [^18^F]fluoride or [^18^F]AlF peak is appeared near the origin (Rf = 0) and Rf of [^18^F]AlF-FAPI-74 is approximately 0.6–0.7

As a result of automated-synthesis with CFN-MPS200, [^18^F]AlF-FAPI-74 was obtained with a radioactivity of 11.3 ± 1.1 GBq at the EOS, and a synthesis time of 31 min on average. The radiochemical yield of [^18^F]AlF-FAPI-74 was 37.0% ± 4.3%, and the residual radioactivity in the QMA cartridge (130 mg with automated synthesis) was 2.3% on average of the total radioactivity (Table 2). The radiochemical purity was ≥ 97% in all formulated solutions at the EOS, AlF-FAPI-74 and sum of chemical impurities including AlF-FAPI-74 in the solutions were 0.3 ± 0.1 µg/mL and 2.8 ± 0.6 µg/mL, respectively (Fig. 4). In the residual [^18^F]fluoride and [^18^F]AlF, there was no peak of ≥ 1% near the origin of the TLC plate at the EOS (Fig. 5). On the other hand, solution of the waste liquid vial after the purification showed that more than 95% of the peaks were located near the origin. The results of radiochemical purity measurements with HPLC up to 4 h after synthesis are shown in Table 3. When saline was used as the formulation solution, a decrease in radiochemical purity was observed over time. Three hours after the first analysis, the radiochemical purity was less than 95%, and after 4 h, the radiochemical purity was clearly degraded (n = 2). In the case of saline containing 100 mg of sodium ascorbate (n = 1), the radiochemical purity was maintained above 95% even after 4 h. However, when saline containing 10 mg ascorbic acid and 90 mg sodium ascorbate (pH 5.0) (n = 1) or commercially available phosphate-buffered saline (pH 7.4) (n = 1) or 10 mM phosphate-buffered saline (pH 6.7) (n = 1) were used as the formulation solution, a gradual decrease in radiochemical purity was observed over time. This was maintained above 95% after 3 h; however, after 4 h, it was nearly 95% or less. In contrast, by using a solution of commercially available phosphate-buffered saline (pH 7.4) containing 100 mg sodium ascorbate (n = 1) or 10 mM phosphate-buffered saline (pH 6.7) containing 100 mg sodium ascorbate (n = 3), it was confirmed that the [^18^F]AlF-FAPI-74 solution was stable with a radiochemical purity of 97%, even following 4 h of synthesis. The residual DMSO and ethanol content calculated from standard solutions of known concentrations were 21 ± 6 ppm and 5.5 ± 0.2 v/v%.

**Table 2:** Distribution of radioactivity after automated-synthesis of [^18^F]AlF-FAPI-74 using CFN-MPS200

Measured point	Radioactivity distribution (%)
Product vial ([^18^F]AlF-FAPI-74)	37.0 ± 4.3
^18^O-Water recovery vial	3.1 ± 1.4
QMA cartridge, 130 mg	2.3 ± 1.3
^18^O-Water waste liquid vial	0.7 ± 1.0
Reactor	8.6 ± 1.5
Dilution vial	0.5 ± 0.2
HLB cartridge	0.3 ± 0.1
Waste liquid vial	45.9 ± 4.0
Formulation vial	0.7 ± 0.4
Synthesis cassette	1.0 ± 0.3

(n = 10)

**Table 3:** Stability of [^18^F]AlF-FAPI-74 for each formulation solution

Formulation solution		Elapsed time after synthesis (hr)			
Batch		0 (EOS)	2	3	4
		Radiochemical purity (%)			
*Saline*					
No. 1		98	96	79	54
No. 2		97	96	94	89
Saline containing 10 mg ascorbic acid and 90 mg sodium ascorbate (pH 5.0)		98	97	96	95
Saline containing 100 mg sodium ascorbate		98	97	97	96
Commercially available phosphate buffered saline (pH 7.4)		98	96	96	95
10 mM phosphate buffered saline (pH 6.7)		98	96	95	89
Commercially available phosphate buffered saline (pH 7.4) containing 100 mg sodium ascorbate		98	97	97	97
*10 mM phosphate buffered saline (pH 6.7) containing 100 mg sodium ascorbate*					
No. 1		98	97	97	97
No. 2		98	97	97	97
No. 3		98	97	97	97

EOS, end of synthesis

## Discussion

In general, the introduction and elution of [^18^F]fluoride into the QMA cartridge were performed from the same side (mainly female side), but in this case, we were able to obtain a high recovery rate of [^18^F]fluoride (90 ± 9% in the manual procedure or residual radioactivity onto the QMA cartridge was 2.3 ± 1.3% with an automated synthesizer) by using a small amount of eluent (0.5 M sodium acetate buffer, pH 3.95 ± 0.05, 300 µL), using the male side for introduction and the female side for elution. In the report by Kersemans et al., elution from a QMA cartridge using 300 µL of 0.5 M sodium acetate was approximately 60% (Kersemans et al. 2018), indicating that this procedure was useful for the recovery of [^18^F]fluoride at low amounts of the eluent. By introducing the eluent from the opposite side of the QMA cartridge, the [^18^F]fluoride trapped in the sorbent near the inlet of the cartridge was prevented from diffusing (or diluting) into the sorbent up to the outlet. Hence, we believe that a high recovery rate could be obtained with a small amount of eluent. In addition, in the radiosynthesis of ^18^F-tracers by CFN-MPS200, VP4 to VP7 were used as lines to connect reagent vials, and we were able to establish a new platform for radiosynthesis by [^18^F]AlF by connecting the QMA cartridge and ^18^O-Water waste liquid vials to these lines.

In the procedure by Giesel et al., fluorination was carried out in two steps (Giesel et al. 2021). Namely, that [^18^F]fluoride collected to the reactor (300 µL) was mixed with 10 mM AlCl_3_ (6 µL) in DMSO (300 µL) and was fluorinated at room temperature for 5 min (production of [^18^F]AlF), then 4 mM FAPI-74 precursor (20 µL) was added to this reactor and was performed a second round of fluorination at 95 ℃ for 15 min (production of [^18^F]AlF-FAPI-74). However, when this manual procedure is transferred to a CFN-MPS200, a two-step reaction must be performed with two reactors (two-pot). Because it is difficult to transfer 20 µL of FAPI-74 precursor solution from the reagent vial to the reactor with high reproducibility, it is necessary to add it to the second reactor in advance. Therefore, the synthesis time will be longer and the loss of radioactivity will be higher because of the multiple steps involved. In addition, The FAPI-74 precursor solution will be added just prior of synthesis to the reactor, which makes it difficult to leak test and flow test in the route of synthesis cassette. We solved this challenge by using a mixed solution of all the reagents for the fluorination. In the fluorination with a mixture of precursor and AlCl_3_ solutions, low radiochemical yields as well as a decrease in radiochemical purity due to radioactive by-products have been reported by McBridge et al. and Kersemans et al. (McBride et al. 2009; Kersemans et al. 2018). In contrast, Allott et al. reported that the addition of 9.5 M ascorbic acid to the reaction mixture during fluorination prevents side reactions and radiolysis (Allott et al. 2017). Therefore, in this study, we added 4 µL of 20% ascorbic acid solution (0.8 mg), and the composition of the other reagents was kept the same as those in manual synthesis because it may affect the labeling rate (D’Souza et al. 2011; Kersemans et al. 2018). In the one-pot and one-step radio-synthesis of [^18^F]AlF-FAPI-74, radiolabeling was performed at 95 °C for 15 min via a reaction at room temperature for 5 min after mixing the [^18^F]fluoride and precursor solution including ascorbic acid, a stable and acceptable radiochemical yield of 37.0 ± 4.3% was obtained, and its radiochemical purity was ≥ 97% at the EOS. The details of the two step synthesis, including its radiochemical yield, radiochemical purity, or synthesis time, have not been reported. However, in addition to the reduction of synthesis time skipping the reagent loading in the one step synthesis, in our results of each manual synthesis, the radiochemical yield (calculated from trapped radioactivity on a QMA cartridge) and radiochemical purity of one step and two step synthesis were comparable (46.0% vs. 49.7 ± 5.3% and ≥ 95). Therefore, a simple procedure and short synthesis time could be achieved without decreasing the radiochemical yield and radiochemical purity.

In the UV chromatogram of the [^18^F]AlF-FAPI-74 solution, two unknown peaks were observed. Although the formation of metal complexes such as [Al^3+^]FAPI-74 and [AlOH^2+^]FAPI-74 has been reported in radiolabeling using [^18^F]AlF (McBride et al. 2010; D’Souza et al. 2011), these were not identified in the present study. However, the total amount of impurities was 2.8 ± 0.6 µg/mL (apparent molar activity is 220 ± 45 GBq/µmol). Even when the maximum injection volume (V) was set to 10 mL, results were sufficient to meet the standard value (50 µg/V) reported by Tshibangu et al. (2020).

In this study, we investigated the stability under various conditions as a proof-of-concept experiment. Regarding the stability of [^18^F]AlF-FAPI-74 with saline as the final formulation solution, the radiochemical purity decreased significantly over time from > 95% at the EOS, which is consistent with previous reports (Boschi et al. 2016; Kersemans et al. 2018). However, as for the large difference in the degree of decomposition between the two batches, 54% and 89%; this difference could not be clarified in this study, although previous reports have also shown the effects of storage temperature and the amount of non-radioactive materials (Kersemans et al. 2018). Reports also showed > 97% after 3 h with 0.2 M acetate buffer (pH 6.8) or less than 5% loss of label with 5 mM phosphate buffer (pH 7.0), respectively. Furthermore, Tshibangu et al. reported that radiolysis could be prevented by adding sodium ascorbate to the formulation solution (Tshibangu et al. 2020). Using these results as a reference, we confirmed the stability of [^18^F]AlF-FAPI-74 with saline containing 10 mg ascorbic acid and 90 mg sodium ascorbate (pH 5.0), saline containing 100 mg sodium ascorbate, and two types of phosphate-buffered saline; the subsequent decrease in radiochemical purity in each solution was slower than that with saline alone. Furthermore, the combination of phosphate-buffered saline and sodium ascorbate showed almost no decrease in purity even after 4 h. In this study, we conducted an in-house clinical study, and checked stability up to 4 h after synthesis. However we plan to check the stability up to 8 h after synthesis, assuming delivery to a remote site, since higher radioactivity of [^18^F]AlF-FAPI-74 is expected to be obtained with large starting activity by extended irradiation time or increased irradiation current.

In this study, we established a one-pot and one-step automated synthesis method using a CFN-MPS200 synthesizer that provides high radioactivity and stable RCP for clinical use. We did not perform endotoxin or sterility tests because we aimed to reflect an optimized method of manual synthesis using the automated synthesizer. Nevertheless, we have previously reported the development of this synthesizer for clinical studies using [^18^F]PSMA-1007 (Naka et al. 2020), and we soon would be able to begin with the corresponding clinical studies.

## Conclusion

We have established a one-pot and one-step automated synthesis method for [^18^F]AlF-FAPI-74 using a CFN-MPS200 synthesizer and have succeeded in producing high radioactivity of more than 10 GBq on average from 32 to 40 GBq of starting activity in a short synthesis time. Furthermore, a stabilized product formulation using phosphate-buffered saline and sodium ascorbate was developed with acceptable quality for human application, and a large number of patients could be inspected in a day. Distribution by satellite concept would also be feasible in areas where delivery is allowed. This method is also expected to be widely used for the selection of therapeutic nuclides for treatment.

## Data Availability

The datasets used and/or analyzed during the current study are available from the corresponding author upon reasonable request.

## Abbreviations

DMSO: Dimethyl sulfoxide
EOS: End of synthesis
FAP: Fibroblast activation protein
GC: Gas chromatography
HPLC: High-performance liquid chromatography
NOTA: 1,4,7-Triazacyclononane-N,N′,N′-triacetic acid
PET: Positron emission tomography
RCP: Radiochemical purity
RT: Retention time
TLC: Thin layer chromatography
UV: Ultraviolet
WFI: Water for injection
Non-radioactive AlF-FAPI-74: AlF-FAPI-74
